# Whole body vibration exercise for chronic low back pain: study protocol for a single-blind randomized controlled trial

**DOI:** 10.1186/1745-6215-15-104

**Published:** 2014-04-02

**Authors:** Xue-Qiang Wang, Yan-Lin Pi, Pei-Jie Chen, Bin-Lin Chen, Lei-Chao Liang, Xin Li, Xiao Wang, Juan Zhang

**Affiliations:** 1 Sport Medicine & Rehabilitation Center, Shanghai University of Sport, Shanghai 200438, China; 2 Department of Rehabilitation Medicine, Shanghai Shangti Orthopedics Hospital, Shanghai 200438, China; 3 Department of Rehabilitation Medicine, Shanghai Punan Hospital, Shanghai 200135, China

**Keywords:** whole body vibration, low back pain, physical therapy, exercise therapy, randomized controlled trial

## Abstract

**Background:**

Low back pain affects approximately 80% of people at some stage in their lives. Exercise therapy is the most widely used nonsurgical intervention for low back pain in practice guidelines. Whole body vibration exercise is becoming increasingly popular for relieving musculoskeletal pain and improving health-related quality of life. However, the efficacy of whole body vibration exercise for low back pain is not without dispute. This study aims to estimate the effect of whole body vibration exercise for chronic low back pain.

**Methods/Design:**

We will conduct a prospective, single-blind, randomized controlled trial of 120 patients with chronic low back pain. Patients will be randomly assigned into an intervention group and a control group. The intervention group will participate in whole body vibration exercise twice a week for 3 months. The control group will receive general exercise twice a week for 3 months. Primary outcome measures will be the visual analog scale for pain, the Oswestry Disability Index and adverse events. The secondary outcome measures will include muscle strength and endurance of spine, trunk proprioception, transversus abdominis activation capacity, and quality of life. We will conduct intention-to-treat analysis if any participants withdraw from the trial.

**Discussion:**

Important features of this study include the randomization procedures, single-blind, large sample size, and a standardized protocol for whole body vibration in chronic low back pain. This study aims to determine whether whole body vibration exercise produces more beneficial effects than general exercise for chronic low back pain. Therefore, our results will be useful for patients with chronic low back pain as well as for medical staff and health-care decision makers.

**Trial registration:**

Chinese Clinical Trial Registry: ChiCTR-TRC-13003708.

## Background

Low back pain (LBP) is a major public health challenge all over the world [1,2]. The lifetime prevalence of LBP is shown to be 84%, and the prevalence of chronic LBP is about 23%, 11 to 12% of people are disabled by LBP [3]. Because of its high prevalence, LBP often leads to a large economic burden with regard to medical expenses and lost wages [4,5]. In the United States, statistics show that total direct and indirect costs for the treatment of LBP are estimated to be more than $100 billion each year [6,7].

Exercise therapy is recommended as an effective treatment for relieving pain and improving back function for patients with LBP by most clinical guidelines [3,8-11]. During the past decade, whole body vibration (WBV) exercise has become increasingly popular for relieving musculoskeletal pain and improving health-related quality of life [12,13]. In whole body vibration, the vibration signals are delivered through a vibratory platform or chair to expose a larger part of the body to the stimulation [14]. WBV exercise consists of standing statically or performing dynamic movements on an oscillating platform. Exercise training using vibratory platforms may be a complementary training to standard physical rehabilitation programs. WBV provides amplitude of displacement (ranging from 0.7 to 14 mm) and a mechanical oscillation of a specific frequency (ranging from 0.5 to 80 Hz) [15,16]. Several studies have demonstrated that whole body vibration exercise can reduce pain for women with fibromyalgia syndrome [17], young patients with musculoskeletal pain [18], and elderly patients with knee osteoarthritis [19].

At present, WBV exercise is being marketed as an intervention for patients with LBP [20-25]. There are two reasons WBV exercise may be useful for alleviating pain in patients with LBP. First, LBP is known to be associated with reduced abdominal and back extensor stabilization muscle activity [26]. It has been proposed that WBV may assist in reducing LBP by activating stretch reflexes and subsequently activating and strengthening the abdominal and back extensor stabilization muscles [23]. Second, LBP is known to be associated with paravertebral muscle spasm, and it has been suggested that WBV at frequencies below 20 Hz may reduce LBP by inducing muscle relaxation [27].

Whole body vibration exercise has a theoretical basis in treatment of LBP, as is evidenced by its clinical use. However, the efficacy of WBV exercise for low back pain is not without dispute. The results of several studies suggest that whole body vibration exercise relieves chronic back pain through a genuine analgesic effect [21-23]. On the other hand, some studies show that WBV exercise was not effective for patients with LBP [28,29], and WBV has even been considered harmful [30].

It is important to ensure that the determination of the effectiveness of WBV exercise for LBP is based on scientific evidence so as not to waste staff time and resources and to avoid unnecessary stress for patients with LBP and their families. Thus, we conduct a single-blind randomized controlled trial (RCT) to determine the effects of WBV for patients with LBP compared to general exercise.

## Methods/Design

### Aim of the study

We will perform an RCT involving whole body vibration exercise in patients with chronic low back pain in order to determine:

1) whether WBV exercise is effective for the treatment of chronic LBP,

2) whether WBV exercise produces more beneficial effects than general exercise for chronic LBP, and

3) adverse effects associated with whole body vibration.

### Study design

**Figure 1:**
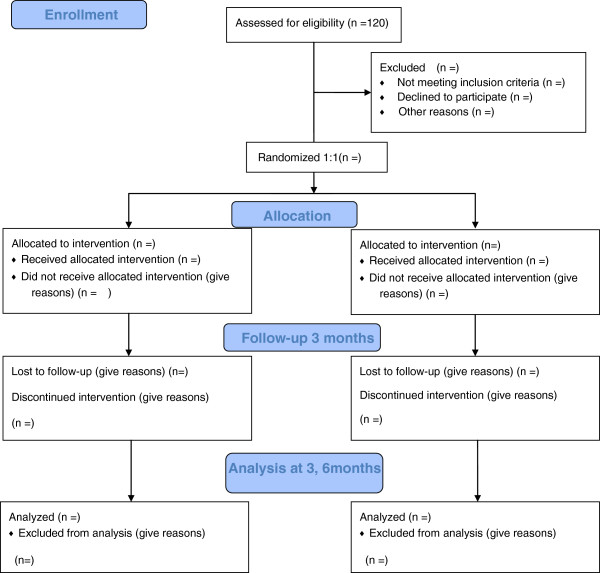
Flow diagram showing whole body vibration exercise for patients with low back pain (LBP).

This is a single-blind randomized controlled trial comparing a WBV exercise with general exercise (Figure 1). A total of 120 patients with chronic LBP will be recruited and investigated from Shanghai Shangti Orthopedic Hospital and Shanghai University of Sport, Shanghai City, China.

All participants, prior to initiation of the study, will accept a questionnaire asking for the following details: basic information (for example, age), past and present job status, medical history, pain (visual analog scale, VAS), back function (Oswestry Disability Index, ODI) and exercise habits (for example, frequency and time/session). Prior to inclusion, informed consent will be sought from all participants in the study.

The subjects will be allocated in a 1:1 ratio by computer-generated randomization if they meet the inclusion criteria. After randomization, patients with chronic LBP will be assigned to the control group (general exercise) or to the intervention group (WBV exercise). The total study period will be 6 months, which has a 3-month intervention and 3-month follow-up period with no intervention. The study will include assessments at the following time points: before intervention, at 3 months, and at 6 months.

### Participants

Inclusion criteria are:

1. age 18 to 60 years old,

2. pain for more than 3 months,

3. lack of abnormal sense and reflection of the lower extremity,

4. medication not expected to change during the study period, and

5. availability 3 times a week over a period of 3 months.

Exclusion criteria are:

Subjects who have any of the following conditions will be excluded:

1. undergone previous surgery, dislocation, fracture, rheumatoid arthritis, and ankylosing spondylitis;

2. severe cardiovascular, progressive neurological deficits, severe osteoporosis, or metabolic diseases;

3. low back pain caused by visceral disease;

4. VAS score (from 1 to 10) above 8;

5. inability to communicate in Chinese;

6. attended WBV exercise last 3 months;

7. pregnant or lactating.

8. uncontrolled hypertension; or

9. other illness, judged by the patient or study physician to make participation in this study inadvisable.

### Withdrawal criteria and management

Patients with chronic LBP will be allowed or be asked to withdraw from the study if:

1. The patient makes such a request.

2. The patient develops a serious disease, such as stroke or heart disease.

3. The patient has an adverse effects related to the WBV exercise.

### Ethical considerations

Before the intervention, each subject will be asked to sign a written informed consent. The study was approved by the ethics committee of the Shanghai University of Sport, China.

### Interventions

Both groups will receive exercise intervention twice a week for 3 months, and each session will involve a warm-up for 5 minutes and cool down for 5 minutes. The participants will be asked to fill out forms to record the times and durations of exercise intervention.

#### Intervention group

**Figure 2:**
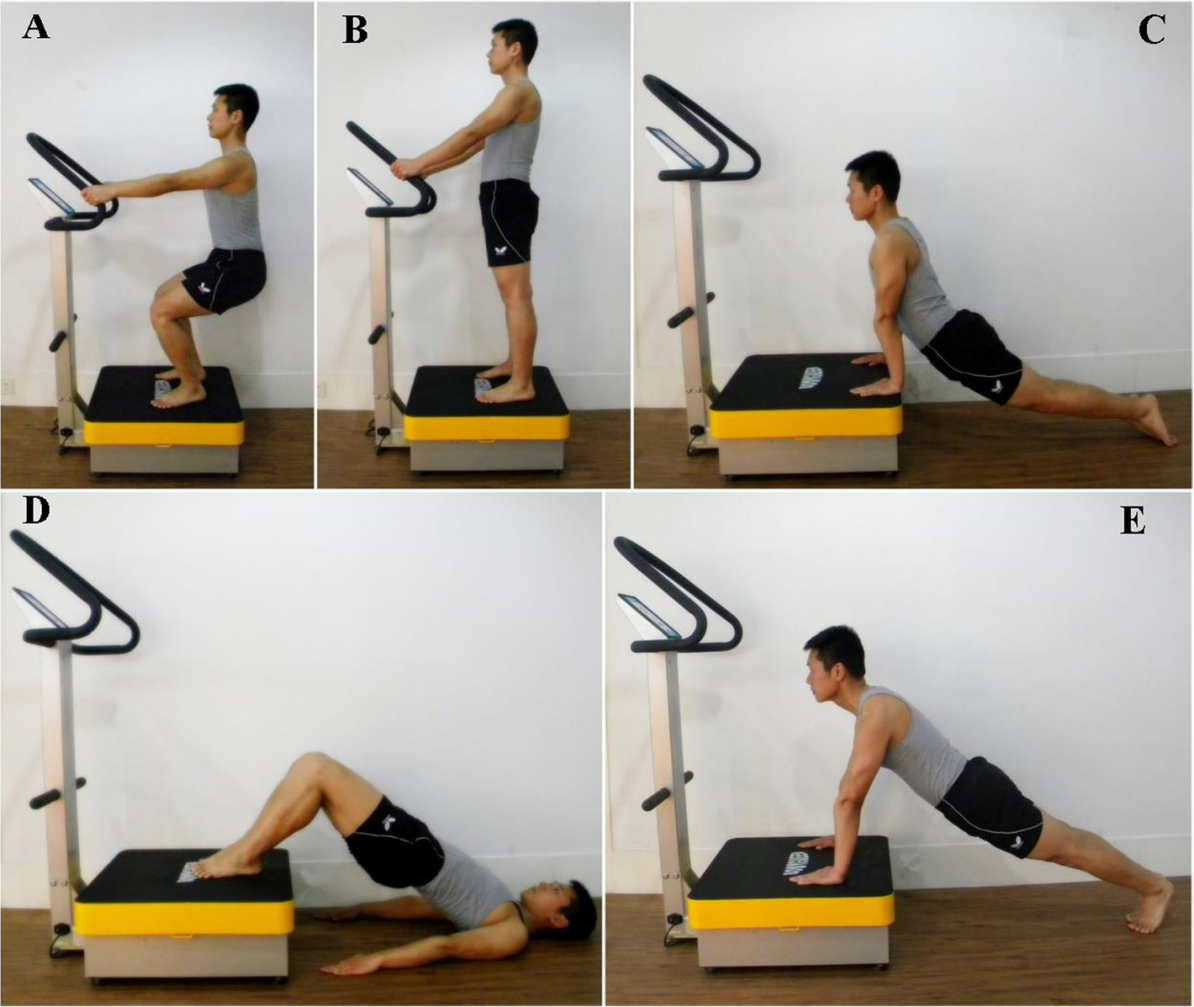
**Illustration of five movements. A**. Double leg stance: place the feet parallel on both sides of machine with heals turned slightly outward. **B**. Deep squat. **C**. Lower back extension. **D**. Bridge pose. **E**. Push up.

All subjects will perform whole body vibration exercise by an available vibration device (AV001; BODYGREEN, Taiwan, China). To eliminate any effect of vibrations, we ask patients to remove their shoes. The WBV exercise protocol includes five exercise positions: double leg stance, deep squat, lower back extension, bridge pose, and push up. More information about the WBV protocol is shown in Table 1 and Figure 2. WBV exercise programs will be led by registered physical therapists.

**Table 1:** Protocol of whole body vibration (WBV) exercise

**Exercise positions**	**Each time (s)**	**Repetitions (n)**	**Frequency (Hz)**	**Interval rest (s)**	**WBV total time (s)**
Double leg stance	90	2	18	30	180
Deep squat	60	2	18	30	120
Lower back extension	90	2	18	30	180
Bridge pose	60	2	18	30	120
Push up	60	2	18	30	120

#### Control group

The participants in the control group will perform the same exercise protocol as the WBV group, but without vibration. The participants in the both groups will be asked to maintain their previous lifestyle and not take part in any other regular rehabilitation programs.

### Outcome measures

Primary outcome measures include the following:

1. Pain intensity, which will be assessed using the VAS. It is usually a horizontal line, 100 mm in length, which is marked with ‘no pain’ (score 0) and ‘unbearable pain’ (score 10) from the left to the right. The patient marks on the line the point that they feel represents their perception of their current state. The amount of pain that a patient feels ranges across a continuum from none to an extreme amount of pain [31].

2. Back function, which will be measured using the Oswestry Disability Index [32], an index that consists of ten sections related to activities of daily living commonly affected by low back pain. Each section is scored from 0 to 5 with higher values indicating more severe impact.

3. Adverse events associated with whole body vibration exercise, which will be reported.

Secondary outcome measures include:

1. Muscle strength and endurance of trunk, which will be tested using a Biodex 3 dynamometer (Biodex Medical Systems, New York, USA). Participants will perform ten maximum concentric contractions for trunk extensors and flexors at the angular velocities of 60°/s, 120°/s. The muscle endurance index is defined as the ratio of the work done during the last five contractions over the first five contractions. Main outcome parameters will be peak torque (Newton-meters), peak torque/ body weight, maximal power output (power) in Watts, work (work) in Joules [33].

2. Trunk proprioception, which will be assessed with a joint position matching test [24]. The subject’s trunk will be moved to reference position (flexion or extension), and maintained at the position for 3 s. Then the subjects will be asked to reproduce the reference position from the neutral position. The subject is instructed to stop a position when he or she thinks that the test position has been reached. The assessor records the position and angle. This sequence of events will be repeated three times, and these three measures were averaged for a score of repositioning error for the first pre-test. The large absolute error is considered as poor proprioceptive acuity.

3. Transversus abdominis activation capacity, which we will assess using the Stabilizer Pressure Biofeedback Unit (PBU, Chattanooga Group, Australia) [34]. The subject will be asked to lie in prone position over a rigid surface, then a PBU device will be placed under the transversus abdominis. The subjects are asked to contract the lower stomach for 10 seconds without moving the back or the hips. Then the assessor will record the pressure reduction from the PBU.

4. Quality of life, which will be assessed with the short form 36 (SF-36) health survey [35]. The SF-36 includes eight scaled scores: vitality, physical functioning, bodily pain, general health perceptions, physical role functioning, emotional role functioning, social role functioning, and mental health. A score of SF-36 is from 0 (worst) to 100 (best).

### Statistical analysis

The SPSS 17.0 and Microsoft Excel 2007 software will be used to perform statistical analyses. Data will be expressed as mean?±?standard deviation (SD). We will use two-way repeated measures analysis of variance (group?×?time) to compare the effect of WBV exercise group with the control group, including the primary and secondary outcomes. If subjects are lost to follow-up, an intention-to-treat analysis will be conducted. A T-test will be used to compare the changes in measures within groups when the analysis of variance reveals a significant difference. Statistical significance is a *P* less than 0.05.

## Discussion

Based on the theory of WBV, it should be an effective treatment for chronic LBP. However, the evidence of WBV is inadequate, and the effect of WBV exercise is disputed for LBP. We will perform a prospective, single-blind, randomized controlled trial of WBV exercise for patients with chronic LBP. We think the study could bring about the proof that WBV may offer an alternative treatment for chronic LBP. WBV exercise could produce more beneficial effects than general exercise for chronic LBP, by reducing pain and improving function and quality of life.

### Strengths and limitations

First, most previous WBV exercise studies have typically ranged in duration from a few weeks to 3 months [20,21,23,24]. The study duration is an intervention period of 3 months and a follow-up period (with no active intervention) of 3 months, giving a total study period of 6 months. Second, in our study, intervention group performs five movements with vibration, but the control group receives the five same movements without vibration for an equal amount of time. Based on this point, the study could reduce other bias compared to previous studies [20-25]. Third, previous studies have mainly focused on pain, back disability, and quality of life. But trunk proprioception and transversus abdominis activation capacity are rarely analysed for WBV exercise on chronic LBP. There are also some limitations in our trial. The total number of subjects involved in the study is not large (120 patients). Moreover, only one vibration frequency (18 Hz) is used to estimate the effect of WBV for chronic LBP; different vibration frequency groups should be added to subsequent studies.

In conclusion, this study aims to determine the effect of WBV exercise for chronic LBP, and to estimate whether WBV exercise produces more beneficial effects than general exercise for chronic LBP. The results of the study will be of benefit to patients, researchers and policy makers with an interest in treatment of LBP.

## Trial status

Patient recruitment is underway.

## Abbreviations

LBP: low back pain; ODI: Oswestry disability index; PBU: pressure biofeedback unit; RCT: randomized controlled trial; SD: standard deviation; SF-36: short form 36; VAS: visual analog scales; WBV: whole body vibration.

## Competing interests

The authors declare that they have no competing interests.

## Authors’ contributions

XQW, YLP and PJC conception and design of the trial. XQW, YLP, PJC, LCL and JZ participated in the trial register, communication and monitoring. BLC, XL and XW participated in the design of statistical analysis. All authors contributed to drafting the manuscript, read and approved the final version.
